# Supplementary material for: Machine learning in Alzheimer’s disease genetics
Source: Nat Commun. 2025 Jul 22;16:6726. doi: 10.1038/s41467-025-61650-z (PMC12280214; doi:10.1038/s41467-025-61650-z)

Supplementary Material: Genome-wide machine learning analysis on Alzheimer's disease

Table of Contents

1. Methods 3

1.1 Data 3

1.2 Gradient boosting machines (GBMs) 8

1.3 Neural networks (NNs) 9

1.4 Model based multifactor dimensionality reduction (MB-MDR) 11

1.5 Genome-wide association study (GWAS) and polygenic risk scores (PRS) 13

1.6 Selection of top predictors 14

1.7 Protein-protein interaction and GO pathway enrichment 18

1.8 Enrichment tests 18

1.9 Interaction testing 19

2. Results 20

2.1 Data splitting 20

2.2 Selection of top predictors in gradient boosting 20

2.3 Selection of top predictors in neural networks 21

2.4 Selection of top predictors in MD-MDR 21

2.5 Pair-wise interaction tests 22

2.6 Comparison of the results with and without APOE region in the same train-test split 22

3. References 25

4. Figures 29

1. Methods

1.1 Data

Data consist of the European Alzheimer & Dementia Biobank (EADB) consortium core sample (EADB-core) which was previously harmonised and made available to collaborators in the EADB consortium (1). Ethical approval for the EADB-core data was obtained separately for each cohort, in accordance with national and institutional requirements. All participants, or their legal guardians, provided written informed consent prior to inclusion. Ethical approval is given in detail by Bellenguez et al (1) – the reported approvals for the EADB-core are summarised below.

EADB-France

- Belgium: approved by the ethics committees of Antwerp University Hospital and the participating neurological centres within the BELNEU consortium, as well as by the University of Antwerp.
- Czech Republic: The Czech Brain Aging Study (CBAS) and CBAS+ were approved by the local ethics committee.
- Denmark: approval obtained as part of The Copenhagen General Population Study (CGPS).
- Finland:
    - ADGEN cohort: Approved by the ethics committee of Kuopio University Hospital (Reference: 420/2016).
    - FINGER study: Approved by the Coordinating Ethics Committee of the Hospital District of Helsinki and Uusimaa (Approval numbers: 94/13/03/00/2009 and HUS/1204/2017).

- France:
    - BALTAZAR study: Approved by the Paris Ethics Committee (Comité de Protection des Personnes Ile de France IV, Saint Louis Hospital).
    - MEMENTO study: Approved by the Comité de Protection des Personnes Sud-Ouest et Outre Mer III (Approval number: 2010-A01394-35).
    - CNRMAJ-Rouen study: Approved by the CPP Ile de France II.
- Italy: Ethics approval is obtained for contribution centres: Brescia, Cagliari, Florence, Milan, Rome, Pertugia, San Giovani Rotondo and Torino
- Spain (France Node): Samples comply with Law 14/2007 and Royal Decree RD 1716/2011.
- Sweden:
    - Uppsala University Hospital: Approved by the ethical committee at Karolinska Institutet or the regional ethical review board.
    - SNAC-K: Approved by the same boards above, with consent obtained from participants or next of kin.
- United Kingdom:
    - MRC Centres (Cardiff, London, Cambridge) and associated sites (SOTON, Nottingham, Manchester, KCL, PRION, CFAS Wales, UCL-DRC): Approved by the respective independent ethics committees of the involved institutions.

EADB-Germany Node

- Germany:
    - Technische Universität München: Approved by the Ethics Committee of the Technical University of Munich, School of Medicine (Approval number: 347-14).

- Göttingen Universität: Approved by the local ethics committee at the University Medical Centre Göttingen.
    - German Dementia Competence Network (DCN): Approved by the respective ethics committees of the 14 participating university hospitals.
    - AgeCoDe Study: Approved by local ethics committees in six German cities.
    - Ethical approval obtained separately for additional contributing studies: DELCODE, VOGEL, Heidelberg/Mannheim, PAGES.
- Greece:
    - HELIAD study: Approved by local ethics authorities; participants provided written informed consent.
- Portugal:
    - Lisbon study: Approved by the local ethics committee.
- Spain (Germany Node):
    - DEGESCO sites (e.g., Fundació ACE, Hospital Clínic IDIBAPS): Conducted under the framework of DEGESCO, which adheres to Spanish biomedical research legislation.
- Switzerland & Austria:
    - Lausanne Study: Approved by the Department of Psychiatry, Geneva University Centre.
    - VITA Study (Austria): Approved by the Ethics Committee of the City of Vienna.

EADB-Netherlands Node

- Netherlands: All studies were approved by the Medical Ethics Committees (METC) of the participating local institutes. Specific cohorts include:

- Erasmus Medical Center: Ethics approval from the local METC.
- Amsterdam Dementia Cohort (ADC): Ethics approval from the VU University Medical Center METC.

Briefly, data from 63 participating cohorts, distributed across 16 countries, were genotyped in three centres, and underwent centralised processing on the EADB University of Lille's Intensive Scientific Computing Mesocentre cluster for quality control. Directly typed variants were used preferentially in our analyses to mitigate potential information loss compared to imputed variants, which can introduce additional noise that may be crucial for sensitive machine learning models. However, known genome-wide significant index variants (see Figure S1, Supplementary Data 1) were taken from imputed data where not present in the genotyped data, to ensure the strongest signals from known loci were present.

Variants were imputed using the TOPMed reference panel, as described previously (1). A final list of 81 genome-wide significant SNPs were extracted using bcftools version 1.14 and the "--regions-file" command. Imputed variants were checked against standard thresholds: Hardy-Weinberg equilibrium (HWE) at $1x10^{-6}$, minor allele frequency (MAF) at 1%, imputation quality at $R^2 > 0.4$, and variant missingness at 2%. All 81 variants passed the MAF filter, having be pre-selected as common variants (see Figure S1 for details), in addition to variant missingness and imputation quality. Two variants failed strict HWE filtering: rs429358 (*APOE*, HWE p = 3.00e-52), and rs7908662 (*PLEKHA1,* HWE p = 3.14e-08). Both were still included in the analysis, as the p-values are still within commonly-used looser HWE p-value thresholds (e.g. (2)), and both SNPs pass a strict HWE threshold run separately in cases (rs429359 p = 1.98e-05; rs7908662 p = 0.0023), and controls (rs429359 p = 0.17;

rs7908662 p = 0.0043), indicating deviations from HWE are mostly explained by differences between cases and controls and therefore acceptable. We note that while the e4-related *APOE* SNP (rs429358) is imputed, rs7412 (*APOE* e2-related) was directly typed, and clinically-derived APOE status is available for the majority of participants. Final counts of participants account for 455 individuals which were excluded due to overlap with data in Kunkle et al (3). For all results which directly assess *APOE*, shown in Figure 3A and 3B, we additionally exclude all participants for whom clinically-derived *APOE* status and imputed *APOE* status (derived from the TOPMed imputed variants only) disagree.

Predictors used as covariates comprise imputed sex, genotyping centre and principal components. Genotyping centre was dummy encoded prior to use as a covariate. The train-test splitting performed prior to analysis was also checked for differences in age-at-baseline between splits and missingness in age-at-baseline. Additional age-related variables were not checked due to high levels of missingness (age at death), availability only in cases (age at last exam), or because they are a mix of age at diagnosis and age at onset (age at onset, also only available in cases) and would not be as informative for ascertaining the suitability of the random split. After the initial random train-test split for internal validation, machine learning models underwent hyperparameter tuning and model selection on the train split using either 4-fold cross-validation (gradient boosting), a further training-validation split of the train set (neural networks), or apparent validation (MB-MDR). Specific procedures for tuning and model selection for each classifier are described in the following sections.

1.2 Gradient boosting machines (GBMs)

Gradient boosting machines (GBMs) fit a forward stage-wise additive model (4), here using shallow trees as weak learners, which are added sequentially to create a strong ensemble learner. A gradient boosting model was fit to the train set and evaluated on the test set. Models were fit using version 1.5.2 of the XGBoost package (5), and the dask package, version 2023.1.1 (6), which allows for distributed training of machine learning models across multiple nodes in a high-performance computing (HPC) cluster. For faster computation, genotypes were pre-shuffled and stored as chunked dask arrays in HDF5 files before handing-off to dask and XGBoost for distributed training. Hyperparameters were tuned on 5000 random participants from the training set using 50 iterations of random search. Number of boosted trees was fixed to 1000 and subsampling-by-tree to 0.7; values for learning rate were drawn from a log-uniform distribution, while max depth and col-sample-by-tree were drawn from a uniform distribution. The GBM was refit to the full training data using the best-performing hyperparameters, and then refit again using only the subset of predictors which had been included in the previous model. This allowed for a reduced predictor space and for computation of importance scores, described below, for all predictors included in the model, which was otherwise unfeasible given available RAM.

To account for covariates in training GBMs, sex and 20 PCs were regressed-off from predictors and the outcome before training models, as recommended in random forests (7). Importance of predictors was assessed using SHapley Additive exPlanatory (SHAP) values (8,9), a game theory-based approach using Shapley values which seek to optimally assign "credit" to predictors. SHAP values are obtained as a single value per individual, per predictor, meaning predicting on an n*p matrix of genotypes results in an n*p matrix of

shap values, where each value represents the impact of the SNP on predictions from the model in that individual. The sum of the SHAP values for a single individual gives the predicted value from the GBM model for that individual. Here we report the mean absolute SHAP values as a summary of the overall impact of a feature on prediction. We note that while SHAP values from a gradient boosted model trained on a binary outcome would normally be interpreted as changes in the log(odds) of the outcome, here they are instead changes in the covariate-adjusted outcome (the residuals after regressing-off confounders), and hence values should not be taken as an exact change in predicted log(odds).

For predictor selection, parsimonious GBM models were sought empirically using the Boruta algorithm (10). The Boruta feature selection algorithm is a common approach to feature selection which combines the raw data with 'shadow features', permuted versions of the original predictors. We apply the approach using the SHAP summary statistics, which performs well compared to alternative SHAP-based approaches (11). Here we re-implement the Boruta-SHAP approach to allow for distributed XGBoost training on dask arrays. Variants which were labelled as important by Boruta-SHAP and passed a 0.0005 threshold for mean absolute SHAP values were taken forward.

1.3 Neural networks (NNs)

To construct neural networks for genetic data analysis, we employed GenNet (12), a command line tool based on Tensorflow (13). GenNet uses a biologically driven configuration in which the connections between the input layer, representing SNPs, and the middle layer, representing genes, are defined using knowledge available in annotation

databases (12). The tool is versatile, supporting various SNP-to-gene connections, and uses ANNOVAR as the default annotation database.

A strength of GenNet is that it allows for insertion of covariates directly into the framework, on the last layer, and hence for a joint prediction of the phenotype from covariates and SNPs (Figure S3). During training, network weights are updated with respect to output from the last layer, which predicts disease status by combining the contributions from genetic data and from the covariates. During prediction in test data, final predictions are further adjusted for covariates to, to capture only the contribution of genetic data. The model's weights are therefore adjusted for covariates during training, but give genetic predictions which are distinct from covariates at run time. For training neural networks, the original train split, which is identical to that used for other methods, was further divided into validation and training, respectively 30% and 70% of the original training set, as iterative *k*-fold cross-validation is unfeasible given the computation times for deep learning models. After tuning hyperparameters on the new training and validation splits, the most performant runs had the following hyperparameters:

- Hidden layer architecture: GO terms-based pathways
- Batch size: 64
- Learning rate: 10^{-3}
- L_1 penalization: 0.08.

For the hidden layers, SNPs were first assigned to genes using ANNOVAR annotations. Every SNP was annotated to exactly one gene, the closest one, as this is the default behaviour in GenNet, except 706 SNPs that were not mapped to any genes and were thus excluded. We

investigated pathways from the Gene Ontology (GO terms) consortium, where pathways are represented hierarchically. Neural network architectures were built with 9 hidden layers, including an initial SNP-to-gene layer, followed by 8 layers where connections between layers progress from local pathways to more general ones as they move deeper through the network. Model hyperparameters for batch size, learning rate (LR) and L_1 (default) penalization were tuned during training. To interpret the contribution of each SNP to the model, we estimated importance scores from the network weights. By starting from the prediction layer and going backwards, step-by-step to the SNP and gene layers, we calculated the importance as the product of the edge weights of each node, both on the SNP and gene level. A SNP influences the prediction through multiple paths, given that the gene to which the SNP is mapped is linked to multiple pathways, and each is connected to various (higher-level) pathways. To summarize the SNP's importance in a given path, we calculated the product of each edge's weight in the path. Then, we took the sum of the importance for each path which involved the target SNP as the SNP's overall importance (Figure S3). A more in-depth explanation of the importance score calculation is in Supplementary section 1.6, in the Neural Network subsection. By starting the calculation on the gene layer, it is also possible to calculate the importance of each gene.

1.4 Model based multifactor dimensionality reduction (MB-MDR)

The MB-MDR method reduces dimensionality in large-scale genetic interaction studies by pooling multi-locus genotypes together according to their association patterns with the phenotype under study (binary, continuous, survival-type). For 2D analyses, it exhaustively explores associations between each pair of SNPs and the phenotype using available-case principles. Three association patterns are identified during dimensionality reduction: low-

risk, high-risk, and indeterminate risk categories of multi-locus genotypes. The final MB-MDR test includes contrasting high and low-risk groups of individuals, per SNP pair. To correct for data snooping and multiple testing, significance is assessed by permutations using a fast implementation of the maxT algorithm (14). The latter has been shown to provide a significance test algorithm that adequately controls the family-wise error rate (FWER) during simultaneous hypothesis testing. MBMDR 4.1.1 runs were parallelized to further speed up analyses. In our analyses, several pre-specified FWER thresholds were compared on the train split $\{0.05, 0.15, 0.25, 0.5, 0.75, 0.8, 0.95, 1\}$, with most the FWER threshold giving the highest AUC during training being $p_{adj} < 1$ (i.e., MB-MDR multiple testing adjusted *p*-values were thresholded to 1, with those below the threshold classed as significant).

The MB-MDR method is non-parametric in the sense that it does not assume specific modes of interaction inheritance. The model-based part of the MB-MDR methodology allows adjusting multi-locus testing for lower-order effects. In particular, 2-locus interaction testing between SNPs A and B are by default model-based, i.e., adjusted for or conditional on possible effects of the component single variants SNP A and SNP B. Singular effects can be modeled via 1 or 2 degrees of freedom, with the latter codominant adjustment being the default so as to remove interference from main effects as much as possible.

Currently, MBMDR software does not accommodate conditional analyses on non-categorical extra covariates. Covariates were therefore regressed off the phenotype and residuals were presented as new phenotype values for MB-MDR analysis. As an association detection strategy, MB-MDR does not readily provide risk predictions for study individuals.

Extraneous routines can be applied with MB-MDR such as described in Gola et al. (15) (MBMDRC) and Le et al. (16) (MRS). MBMDRC takes, on the training set, the significant SNP pairs and SNP. Then, in each cell of the 3*3 matrix (or 3*1 vector for single SNP), calculates the phenotype's mean (or the proportion case/control in the binary case) of all the individuals in the train set with that particular SNP-SNP configuration. Furthermore, on the test set, for each individual, MBMDRC calculates the risk score as the average of the phenotype’s mean in the cell of the 3*3 matrix (3x1 for MB-MBDR 1d) for the various cells the individual is part of. We used the approach outlined in Gola et al. (15) in all analyses.

1.5 Genome-wide association study (GWAS) and polygenic risk scores (PRS)

To allow for fair comparison between machine learning approaches and standard GWAS/PRS methods, a GWAS was performed on the train-split of the EADB-core dataset (29,180 individuals comprising 14,006 cases and 15,174 controls). Linear additive models were fit using the --glm option in PLINK2 (v2.00a3.3LM) with sex (binary), genotyping centre (3 categories) and 20 principal components as covariates. Covariates were standardised using --covar-variance-standardize, with genotyping centre handled as a categorical covariate using “--split-cat-pheno omit-most covar-01 genotyping_center”.

PRS were generated with summary statistics from the train-set GWAS, to ensure comparability with machine learning approaches, using the Bolt-Predict method implemented in LDAK version 5.2, as this is recommended as the best-performing option in LDAK (supplementary Table 1) when raw genotypes are available for the training data (17). LDAK Bolt-Predict was implemented using standard settings recommended in the

documentation (18). The procedure was run separately to generate PRS with and without the *APOE* region, e.g.:

**Step**	**Commands**
Pre-processing	- Liftover bed/bim/fam files to GRCh37 using the UCSC genome browser liftover function, and change from rsids to chr:pos IDs<br>- Convert summary statistics to "Predictor, A1, A2, n, Z" format<br>- Write a random shuffle of 5k individual IDs for use in training using "shuf" and "awk"
Get annotations	- wget --no-check-certificate https://genetics.ghpc.au.dk/doug/bld.zip<br>- unzip bld.zip
Get weightings	- ldak5.2.linux --cut-weights sections --bfile without_apoe_for_ldak --no-thin YES<br>- ldak5.2.linux --calc-weights-all sections --bfile without_apoe_for_ldak --max-threads 5<br>- mv sections/weights.short bld65
Get taggings	- ldak5.2.linux --calc-tagging bld.ldak --bfile without_apoe_for_ldak --ignore-weights YES --power -.25 --annotation-number 65 --annotation-prefix bld --window-kb 1000 --save-matrix YES --max-threads 5 --keep ../random_5k_subsample_for_h2matrix.txt<br>- *Note that --keep is used as the docs note: "Note that if you are analysing individual-level data for tens of thousands of samples, it is not necessary to use all of these when calculating the tagging file and heritability matrix. Instead we suggest using --keep <keepfile> to specify 5000 randomly-selected samples, which will substantially reduce computational demands."*
Get SNP heritability	- ldak5.2.linux --sum-hers bld.ldak --tagfile bld.ldak.tagging --summary ../grc37_sumstats_for_ldak.txt --matrix bld.ldak.matrix --max-threads 5
Get effect sizes	- ldak5.2.linux --bolt bolt --pheno without_apoe_for_ldak.pheno --bfile without_apoe_for_ldak --ind-hers bld.ldak.ind.hers --cv-proportion .1 --max-threads 5
Calculate PRS	- ldak5.2.linux --scorefile bolt.effects --bfile without_apoe_for_ldak --power 0 --calc-scores without_apoe_for_ldak_test --max-threads 5

Supplementary Table 1: LDAK pipeline commands.

1.6 Selection of top predictors

For all ML methods, the top SNP selection process outlined below was applied to the initial train-test split. The same numerical cut-off of number of SNPs was then applied to all other

random splits, with the exception of MB-MDR, for which the *p*-value threshold was applied to all.

Gradient boosting

Top predictors were chosen empirically by selecting the right tail of the distribution of mean absolute SHAP values, following the "elbow method" to visually identify a hinge point in the distribution. There is no pre-determined threshold at which SHAP values can be taken as meaningful (9), though cut-offs such as the top 1% have been used. Here we use a combination of graphically checking the right tail of the distribution, and Boruta-based feature selection (10) to highlight SNPs which have the greatest impact on model predictions.

Boruta-based feature selection was performed using a distributed implementation of the Boruta-SHAP method, which can be found at https://github.com/seafloor/daxos. Briefly, this entails inserting unassociated "shadow" features into the data and determining whether, on average, genuine predictors are more important to the model than these noise variables. Importance to the model is determined here by mean absolute SHAP value. While an implementation of this is available (Boruta-SHAP, https://github.com/Ekeany/Boruta-Shap), it uses a non-distributed scikit-learn based API, which would not compute on available memory for this dataset on our servers. Furthermore, it does not implement the adapted BorutaPy method (https://github.com/scikit-learn-contrib/boruta_py) which includes corrections for multiple testing which are likely to be more useful in high-dimensional biological data. Here we adapt the BorutaPy approach from Daniel Homola to handle distributed training in dask and use of the mean absolute SHAP value in feature selection. Our distributed Boruta-SHAP approach, applied to gradient boosting trained with

the *APOE* region, was run with default parameters, 10 iterations, the default two-step multiple testing procedure, and using the 99th percentile of the shadow features to determine importance. Results showed 937 SNPs as important, which included all 108 SNPs from the extreme tail of the distribution of mean absolute SHAP values, and 435 tentative SNPs. Only SNPs marked as "important" were taken forward.

Neural networks

The network is composed of the SNP input layer, a gene layer, and 8 layers of biological pathways, extracted from GO. The selected GO terms are not limited to the biological process, but include molecular function, biological process, and cellular components. Moreover, all GO terms were considered, not only the AD-specific ones.

GO terms directly influenced by genes (i.e., local pathways) constitute the first biological pathway layer. Genes are mapped to the local pathways, and an extra node on the pathway layer is created to group all the genes that are not mapped to any local pathway. Moreover, the weights of the connections between genes and pathways are randomly initialized and learned from the data, thus not influenced by previous research.
Finally, 214,487 SNPs constituted the input feature set (out of 215,193 in total), barring only 706 SNPs that the default ANNOVAR model could not map to any gene. Hence, there is no bias toward exonic variants.

To calculate the importance scores, we used a recursive procedure. Firstly, we calculated the importance of the second-to-last layer, i.e., the last pathway layer. We define the importance for a generic node i in the second-to-last layer as IS_i^{p-1} .

Said IS_i^{p-1} is the absolute value of the edge (link) between the pathway and the prediction, namely $IS_i^{p-1} = \left|e_{i1}^{(p-1)}\right|$, connecting the i-th pathway to the prediction (the final layer has only one node: the prediction node). Then, for a generic node in the third-to-last layer, representing a local pathway in GO approaches, we calculated the importance of the node as follows:

$$IS_i^{p-2} = \Sigma_{j:e_{ij}^{(p-2)} \neq 0} \left| e_{ij}^{(p-2)} \cdot IS_j^{p-1} \right| .$$

With e_{ij}^{p-2}, the link between nodes i of the $p-2$ layer and node j of the $p-1$ layer. Hence, the IS is the weighted sum of the importance of all the pathways to whom the target node is connected, with the edge weight as weight. Finally, the last step of the recursive procedure calculates the importance of each SNP as the weighted sum of the importance of all the genes the SNP is connected to. Hence, in formula, for a generic SNP i: $IS_i^1 = \Sigma_{j:e_{ij}^{(1)}\neq 0} \left| e_{ij}^{(1)} \cdot IS_j^2 \right|$. Moreover, to better visualize the IS and without losing generality, we scaled the ISs in the SNP layer, such that the SNP with the higher importance has an IS of 1.

To identify the relevant hits, we estimate a Gaussian distribution based on the importance scores of all the SNPs. We are interested in the importance scores (hence in the SNPs) that are outliers in the estimated distribution, I.e., that are too high (hence, the right tail) to fit into the same distribution as all the SNPs. For a target SNP, we tested the null hypothesis that the importance come from the Gaussian distribution estimated on all the SNPs: H_0: $IS_i^1 \sim N\left(E(IS^1); SD(IS^1)\right)$. Hence, the *p*-value we calculated represents how unlikely it is that the target importance score comes from that distribution.

MB-MDR

Significant MB-MDR SNPs (MB-MDR 1D) and SNP pairs (MB-MDR 2D) were based on maintaining the adjusted *p*-value (p_{adj}) < 1, for each dimension (1D and 2D). Several MB-MDR *p*-value cut-offs were considered $\{0.05, 0.15, 0.25, 0.5, 0.75, 0.8, 0.95, 1\}$ when constructing MB-MDRC 1d and MB-MDRC 2d, with larger cut-offs leading to more terms in the respective scores. The final prediction reported comes from the most lenient threshold (p_{adj} < 1).

1.7 Protein-protein interaction and GO pathway enrichment

The publicly available tool STRING v12.0 (19) was used to determine protein-protein interaction and gene ontology pathway enrichment for the top annotated genes from the hits obtained by GBMs, MB-MDR and NNs. Settings for this analysis included: full STRING network with interaction sources determined using default options; thresholding for a minimum required interaction score of medium confidence, 0.4 (Figure S9). In total, 53 edges were found, more than a four-fold increase from the expected number of edges (13).

1.8 Enrichment tests

Formal enrichment tests were performed using microglial (21), astrocytic (22), and synaptic (23) regions. Astrocyte regions are described in Endo et al. (22) as genes showing enrichment in astrocytes across brain regions in mice. After mapping to human genes using ensembl's BioMart server and restricting to unique ensemble IDs, we obtained a list of 757 astrocyte-enriched genes. For the synapse, genes were obtained from the SynGo portal (23). These include 1,535 genes annotated to the synapse. For microglial regions, data taken from

Gosselin et al. (21); we obtained a list of 761 genes enriched in microglia. Lists of genes can be found at https://github.com/seafloor/escott-price-lab-pipelines/tree/main/output/pathways. Code for processing gene lists directly from publications can be found at https://github.com/seafloor/escott-price-lab-pipelines/tree/main/workflows/pathways.

For all genes, gene boundaries were obtained by querying ensembl using the R biomaRt package, and all regions to be searched were then expanded to include 35kb upstream and 10kb downstream of the gene. To obtain *p*-values, a bootstrapping approach was undertaken. Here, for a given list of SNPs from a machine learning analysis, the observed number of matches to regions was first calculated. Then, regions of equal number and size to those in the search list were randomly selected from the genome, and the number of matches calculated. This procedure was performed 10,000 times to generate a null distribution. Finally, the proportions of top SNPs in the randomly selected regions were compared with the proportion of top SNPs in the actual microglia and astrocytes gene lists. The number of times when the proportion of top SNPs in the bootstrapped regions was higher than in the actual gene lists were counted, then divided by the number of simulations, and reported as *p*-values for enrichment.

1.9 Interaction testing

Interactions were tested between all SNPs reported in Tables 1 and 2. Taking all pairwise combinations between top SNPs within models, 17,205 unique logistic regression models with main effects and interactions terms were fit, including sex, genotyping centre and principal components as covariates. Reported *p*-values are for the interaction term in the

regression model. Results are adjusted for multiple testing using a Benjamini-Hochberg FDR correction (at $p = 0.05$).

2. Results

2.1 Data splitting

Data were exported to additively encoded raw files from PLINK2 (--export A) before being randomly shuffled and then split into train and test data. Splits were evaluated for similarities across standard demographic information which was available in the data (Figure S2). Tests of categorical variables (chi-square) between the train and test split, as well as for missingness in age-at-baseline variables, also showed no significant differences between the two splits (data not shown).

2.2 Selection of top predictors in gradient boosting

See section 1.6 for a description of how predictor selection was applied across train-test splits. After visual inspection of the distribution of mean absolute values we investigated a 0.0005 threshold (around 1.3%) as this denoted the extreme tail of the distribution (Figure S4a). All 108 of the SNPs meeting this threshold were labelled as important by the Boruta method. By comparison, a less extreme threshold of 5% (Figure S4b) would include a larger set of 406 variants, but only around 85% of these were marked as important by Boruta feature selection. The 0.0005 threshold therefore marks a reasonable cut-off for top SNPs which can be taken forward for further investigation. The same threshold was applied to XGBoost models trained with and without the *APOE* region.

2.3 Selection of top predictors in neural networks

In Neural networks, we utilized the default ANNOVAR annotations present in the GenNet package to link SNP-genes. 214,487 SNPs SNPs were used in selection of top predictors (out of 215,193 in total) including all SNPs mapped to a gene in ANNOVAR. 706 SNPs were not mapped to genes in the initial SNP-to-gene layer and could not be used in the model or calculation of importance scores.

We considered SNPs as top predictors if they have a Bonferroni adjusted p-value lower of 0.05, meaning that they are extremely unlikely to be modelled by the Gaussian distribution estimated on all the importance scores (Figure S5).

2.4 Selection of top predictors in MD-MDR

Exploring different significance thresholds for SNPs inclusion using grid search {0.05, 0.15, 0.25, 0.5, 0.75, 0.8, 0.95, 1} in the train split only, the prediction accuracy performance, as measured by AUC, suggested a slight improvement by loosening the p_{adj}-value threshold, with the best performance attained with a threshold of $p_{adj} < 1$. Thus, we considered SNPs with an adjusted *p*-value lower than 1 as top predictors.

The significant SNP-SNP interaction found by MB-MDR are depicted in Figure S7. An edge means that there is at least one significant SNP-SNP interaction between SNPs that map to those genes, while the node size is proportional to the degree of the node. Self-loops represent significant SNP-SNP interaction between SNPs of the same gene. In panel S5A, we grouped all the genes in the APOE region (depicted in blue), showing no interaction between those genes and the rest. At the same time, on panel S5B, we decomposed the interactions in the APOE region into the various genes.

2.5 Pair-wise interaction tests

From 17,205 unique pairwise SNP-SNP interaction tests, 13 were significant after Benjamini-Hochberg FDR correction (at p = 0.05) for multiple testing and removal of all 137 SNP-SNP pairs which involved SNPs which were both located within the *APOE* region. Association with the *APOE* region is likely due to the high linkage disequilibrium between variants in the region. Taking the initially significant interaction between rs429358 and rs72654473 (p_{FDR} = 1.36e^{-6}) within the *APOE* region as an example, we note that while rs429358 and rs72654473 are in approximate LE in our data (r^2 = 0.03, D` = 0.26), rs72654473 is in moderate to high LD with rs7412 ($r^2$ = 0.52, $D$` = 0.99), and an extended logistic regression model which also includes rs7412 as a main effect in the model (i.e. y ~ rs429358 + rs72654473 + rs7412 + rs429358:rs72654473 + covariates) shows no significant effects for an interaction between rs429358 and rs72654473 (p = 0.91).

13 SNP-SNP pairs which passed significance thresholds (BH-FDR p = 0.05) involved at least one SNP outside the *APOE* region. Of these, only 2 survived more stringent correction for multiple testing (FDR p = 0.01), or a severe Bonferroni correction. Both pairs correspond to SNPs in the *APOE* and *MS4A** regions, as do many of the 13 SNP pairs which passed FDR (p = 0.05) correction.

2.6 Comparison of the results with and without APOE region in the same train-test split

ML models were compared when trained with and without the *APOE* region on the initial train-test split, allowing for direct comparison across approaches. As noted in the main text, models trained with *APOE* were both highly correlated (between r=0.80 and r=0.87) and highly performant (maximum AUC from gradient boosting, AUC=0.692). Correlations and

AUCs were markedly lower for models trained without the *APOE* region, as expected; distributions of predictions from these models were unimodal and did not stratify by *APOE* status (Figure S9).

GBM trained without the *APOE* region highlighted additional known loci at *MME* and *TPCN1*. Neural networks (NN) highlighted known loci in *APOE, BIN1, CYP27C1, ABCA7, APP, CLU*, and *EGFR*. In this train-test split, four additional novel loci identified by NNs were potentially associated with AD: *THBS1, SOD1*, *and PCSK9*. SNPs prioritised by GBMs showed significant enrichment in microglial regions when trained with the *APOE* region (n = 108 SNPs; p=0.0007) and without (n = 140 SNPs; and p=0.015). SNPs identified by gradient boosting without the *APOE* region had slightly stronger evidence for enrichment in astrocytes than the with-*APOE* model (p=0.006 and p=0.011 respectively). GBMs and MB-MDRC 1d showed significant microglial enrichment (p=0.015, p=0.046 respectively) without the *APOE* region, while only NNs and MBMDRC 1d, both with *APOE* included, were enriched for synaptic regions (p=0.03, p=0.03 respectively; see Supplementary Table 2, and Supplemental Figure S11).

Supplementary Table 2: Bootstrap enrichment analysis for genes enriched in astrocytes, microglia and synapses, with 10,000 repetitions.

**Method**	**Astrocyte**	**Microglia**	**Synapse**
MBMDR APOE 2d	0.0330	0.0183	0.0650
MBMDR APOE 1d	0.0312	0.0063	0.0286
MBMDR noAPOE 1d	0.0149	0.0457	1.0000
GenNet APOE	0.0012	0.0068	0.0281
XGB APOE	0.0111	0.0007	0.3730
XGB noAPOE	0.0060	0.0148	0.4140

The top SNPs from GBMs trained without the *APOE* region showed a greater enrichment in astrocytes and had a greater overlap with the previously reported GWAS hits than the model including the *APOE* region. The enrichment of the SNPs prioritised by GBM in astrocytes in the model without the *APOE* region may suggest a different disease development mechanism in the absence of *APOE*-e4 related risk. However, we note that comparisons here are restricted to a single train-test split. In addition, Supplemental Figure S11:A presents a clustermap of scaled SHAP values from GBMs run on this train-test split (including *APOE* region). In this figure each row is an individual and each column is a SNP, where each column is separately scaled. The figure clearly highlights clusters of individuals characterised by different sets of SNPs.

3. References

1. Bellenguez C, Küçükali F, Jansen IE, Kleineidam L, Moreno-Grau S, Amin N, et al. New insights into the genetic etiology of Alzheimer's disease and related dementias. Nat Genet 2022 544 [Internet]. 2022 Apr 4 [cited 2023 Jan 24];54(4):412–36. Available from: https://www.nature.com/articles/s41588-022-01024-z
2. Canela-Xandri O, Rawlik K, Tenesa A. An atlas of genetic associations in UK Biobank. Nat Genet 2018 5011 [Internet]. 2018 Oct 22 [cited 2024 Apr 17];50(11):1593–9. Available from: https://www.nature.com/articles/s41588-018-0248-z
3. Kunkle BW, Grenier-Boley B, Sims R, Bis JC, Damotte V, Naj AC, et al. Genetic meta-analysis of diagnosed Alzheimer's disease identifies new risk loci and implicates Aβ, tau, immunity and lipid processing. Nat Genet 2019 513 [Internet]. 2019 Feb 28 [cited 2021 Jul 26];51(3):414–30. Available from: https://www.nature.com/articles/s41588-019-0358-2
4. Friedman JH. Greedy Function Approximation: A Gradient Boosting Machine [Internet]. Vol. 29, The Annals of Statistics. Institute of Mathematical Statistics; 2001 [cited 2018 Feb 14]. p. 1189–232. Available from: http://www.jstor.org/stable/2699986
5. Chen T, Guestrin C. XGBoost. In: Proceedings of the 22nd ACM SIGKDD International Conference on Knowledge Discovery and Data Mining - KDD '16 [Internet]. New York, New York, USA: ACM Press; 2016 [cited 2018 Feb 14]. p. 785–94. Available from: http://dl.acm.org/citation.cfm?doid=2939672.2939785
6. Rocklin M. Dask: Parallel Computation with Blocked algorithms and Task Scheduling. PROC 14th PYTHON Sci CONF [Internet]. 2015 [cited 2023 Jan 24]; Available from:

https://www.youtube.com/watch?v=1kkFZ4P-XHg

7. Zhao Y, Chen F, Zhai R, Lin X, Wang Z, Su L, et al. Correction for population stratification in random forest analysis. Int J Epidemiol [Internet]. 2012 Dec 1 [cited 2018 Jun 25];41(6):1798–806. Available from: https://academic.oup.com/ije/article-lookup/doi/10.1093/ije/dys183

8. Lundberg SM, Allen PG, Lee S-I. A Unified Approach to Interpreting Model Predictions. Adv Neural Inf Process Syst [Internet]. 2017 [cited 2023 Jan 24];30. Available from: https://github.com/slundberg/shap

9. Lundberg SM, Erion G, Chen H, DeGrave A, Prutkin JM, Nair B, et al. From local explanations to global understanding with explainable AI for trees. Nat Mach Intell 2020 21 [Internet]. 2020 Jan 17 [cited 2023 Jan 24];2(1):56–67. Available from: https://www.nature.com/articles/s42256-019-0138-9

10. Kursa MB, Rudnicki WR. Feature selection with the boruta package. J Stat Softw. 2010 Sep 16;36(11):1–13.

11. Verhaeghe J, Van Der Donckt J, Ongenae F, Van Hoecke S. Powershap: A Power-full Shapley Feature Selection Method. 2022 Jun 16 [cited 2023 Jun 23];71–87. Available from: http://arxiv.org/abs/2206.08394

12. van Hilten A, Kushner SA, Kayser M, Arfan Ikram M, Adams HHH, Klaver CCW, et al. GenNet framework: interpretable deep learning for predicting phenotypes from genetic data. Commun Biol 2021 41 [Internet]. 2021 Sep 17 [cited 2023 May 2];4(1):1–9. Available from: https://www.nature.com/articles/s42003-021-02622-z

13. Abadi M, Barham P, Chen J, Chen Z, Davis A, Dean J, et al. TensorFlow: A system for large-scale machine learning [Internet]. 2015 [cited 2018 Jun 18]. Available from: https://www.tensorflow.org/

14. Lishout F Van, Gadaleta F, Moore JH, Wehenkel L, Steen K Van. gammaMAXT: a fast multiple-testing correction algorithm. BioData Min [Internet]. 2015 Nov 20 [cited 2024 Feb 7];8(1). Available from: https://pubmed.ncbi.nlm.nih.gov/26594243/

15. Gola D, König IR. Empowering individual trait prediction using interactions for precision medicine. BMC Bioinformatics [Internet]. 2021 Dec 1 [cited 2021 Apr 21];22(1):74. Available from: https://bmcbioinformatics.biomedcentral.com/articles/10.1186/s12859-021-04011-z

16. Le TT, Gong H, Orzechowski P, Manduchi E, Moore JH. Expanding polygenic risk scores to include automatic genotype encodings and gene-gene interactions. Bioinforma 2020 - 11th Int Conf Bioinforma Model Methods Algorithms, Proceedings; Part 13th Int Jt Conf Biomed Eng Syst Technol BIOSTEC 2020. 2020;79–84.

17. Zhang Q, Privé F, Vilhjálmsson B, Speed D. Improved genetic prediction of complex traits from individual-level data or summary statistics. Nat Commun 2021 121 [Internet]. 2021 Jul 7 [cited 2023 Jan 24];12(1):1–9. Available from: https://www.nature.com/articles/s41467-021-24485-y

18. Speed D. LDAK Bolt-Predict [Internet]. [cited 2022 Oct 1]. Available from: https://dougspeed.com/bolt-predict/

19. Szklarczyk D, Kirsch R, Koutrouli M, Nastou K, Mehryary F, Hachilif R, et al. The STRING database in 2023: protein-protein association networks and functional enrichment analyses for any sequenced genome of interest. Nucleic Acids Res [Internet]. 2023 Jan 6 [cited 2024 Apr 25];51(D1):D638–46. Available from: https://pubmed.ncbi.nlm.nih.gov/36370105/

20. Harris CR, Millman KJ, van der Walt SJ, Gommers R, Virtanen P, Cournapeau D, et al. Array programming with NumPy. Nature [Internet]. 2020 Sep 17 [cited 2020 Sep

23];585(7825):357–62. Available from: http://www.nature.com/articles/s41586-020-2649-2

21. Gosselin D, Skola D, Coufal NG, Holtman IR, Schlachetzki JCM, Sajti E, et al. An environment-dependent transcriptional network specifies human microglia identity. Science (80-). 2017 Jun 23;356(6344):1248–59.

22. Endo F, Kasai A, Soto JS, Yu X, Qu Z, Hashimoto H, et al. Molecular basis of astrocyte diversity and morphology across the CNS in health and disease. Science (80-) [Internet]. 2022 Nov 4 [cited 2024 Jan 10];378(6619). Available from: https://www.science.org/doi/10.1126/science.adc9020

23. Koopmans F, van Nierop P, Andres-Alonso M, Byrnes A, Cijsouw T, Coba MP, et al. SynGO: An Evidence-Based, Expert-Curated Knowledge Base for the Synapse. Neuron [Internet]. 2019 Jul 17 [cited 2024 Jan 10];103(2):217-234.e4. Available from: https://pubmed.ncbi.nlm.nih.gov/31171447/

24. Andrews SJ, Fulton-Howard B, Goate A. Interpretation of risk loci from genome-wide association studies of Alzheimer's disease. Lancet Neurol. 2020 Apr 1;19(4):326–35.

25. Wightman DP, Jansen IE, Savage JE, Shadrin AA, Bahrami S, Holland D, et al. A genome-wide association study with 1,126,563 individuals identifies new risk loci for Alzheimer's disease. Nat Genet 2021 539 [Internet]. 2021 Sep 7 [cited 2021 Nov 25];53(9):1276–82. Available from: https://www.nature.com/articles/s41588-021-00921-z

4. Figures

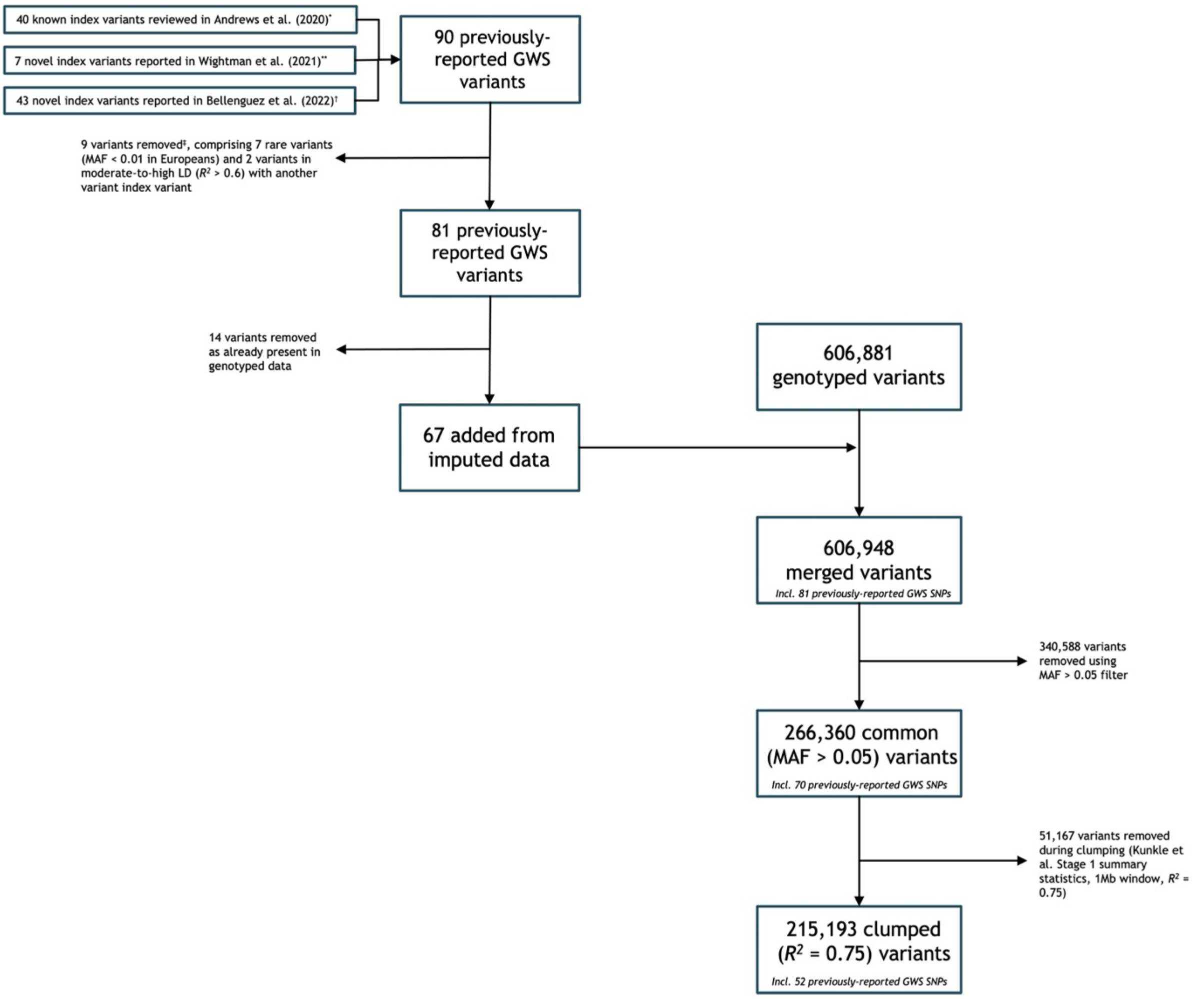

Figure S1: pipeline for processing genotyped and imputed data. 81 variants which have been previously reported as genome-wide significant in large meta-analyses were extracted from imputed data and merged with the genotype data before QC, where 14 variants of the GWS SNPs were already present in the genotyping data. *40 Known variants taken from Table 1 of the review by Andrews et al. (24), where a random variant was select from each locus where multiple GWS SNPs have been reported in high LD with each other. **7 novel variants were extracted from Table 1 in Wightman et al. (25). †43 novel variants were extracted from Table 2 in Bellenguez et al. (1), excluding rs871269 (*TNIP1*), which is present in Wightman et al. ‡9 variants were removed before merging with the genotyped data, 7 of

which were due to low allele frequency (MAF < 0.01 in 1000 genomes Europeans subset): rs141749679 (*SORT1*), rs143080277 (*NCK2*), rs184384746 (*HESX1*), rs187370608 (*TREM2*), rs7185636 (*IQCK*), rs114360492 (*CNTNAP2*), rs113020870 (*AGRN*). A further 2 were removed due to being part of a pair of SNPs in LD, where the SNP with the lowest reported p-value was kept: rs708382 (*GRN*) removed, rs5848 (*GRN*) retained; rs5011436 (*TMEM106B*) removed, rs13237518 (*TMEM106B*) retained. After merging and QC, 52 variants from the previously-reported GWS SNP list were present, 45 of which were added from imputed data, and 7 of which were already present in the genotyping data.

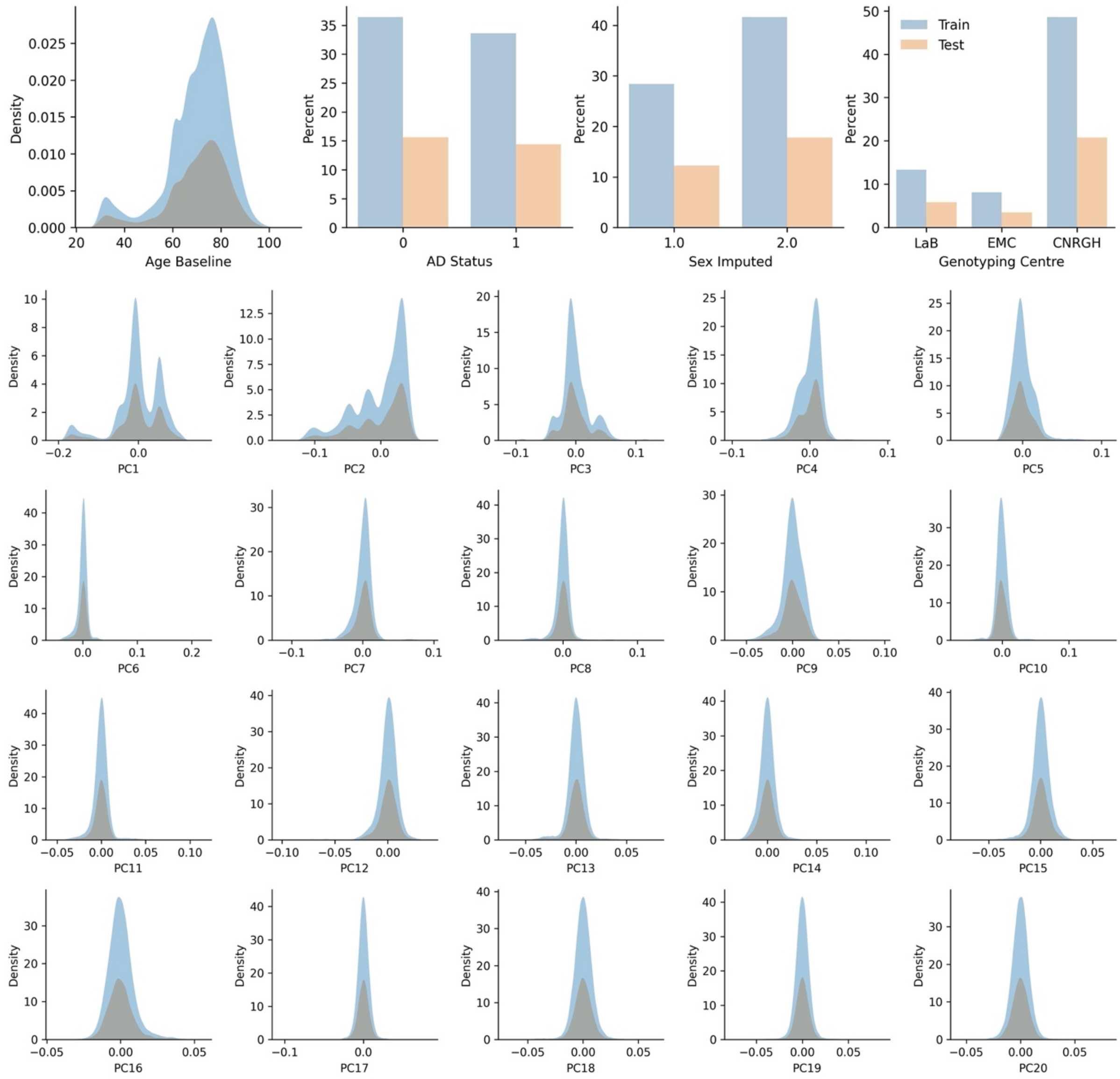

Figure S2: Distribution of demographic variables across a single (the initial) train-test split. Bars and density plots for the train split are shown in blue, and for the test split in red. All 20 principal components are shown unstandardised. Similar consistency for train and test data is visible across additional random splits.

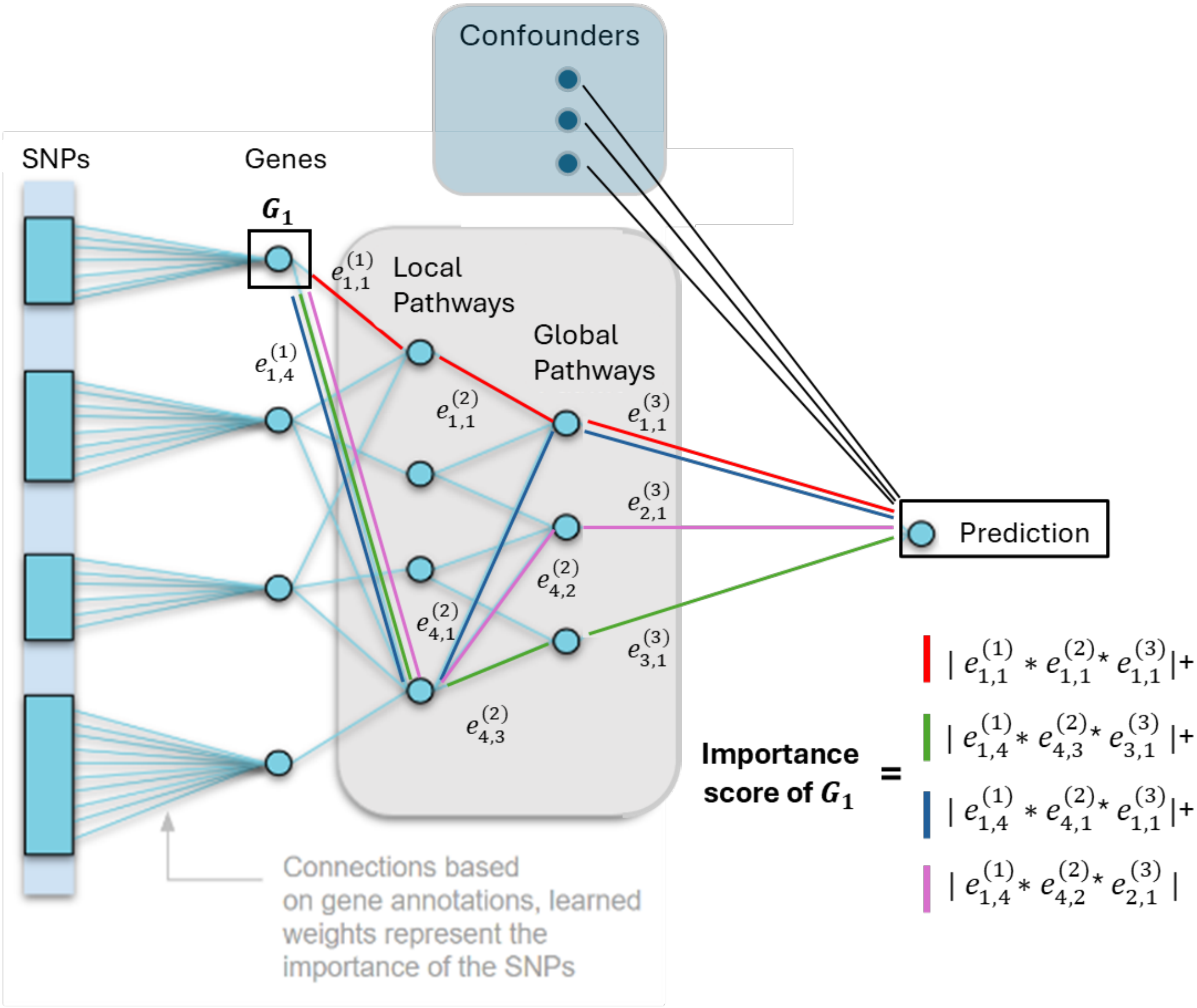

Figure S3: The structure of the GenNet neural network is showcased. SNPs are mapped through ANNOVAR to the closest gene; then, genes are involved in multiple local pathways (based on GO annotations) that are associated with higher-order pathways. Finally, said pathways, together with the confounders, predict Alzheimer's status. A generic $e^{(k)}_{ij}$ represents the edge weight connecting node i of the k layer to node j of the $k+1$ layer. The importance score of the first gene is calculated in the figure as the sum of the product of each path, highlighted in different colour for visualization purposes.

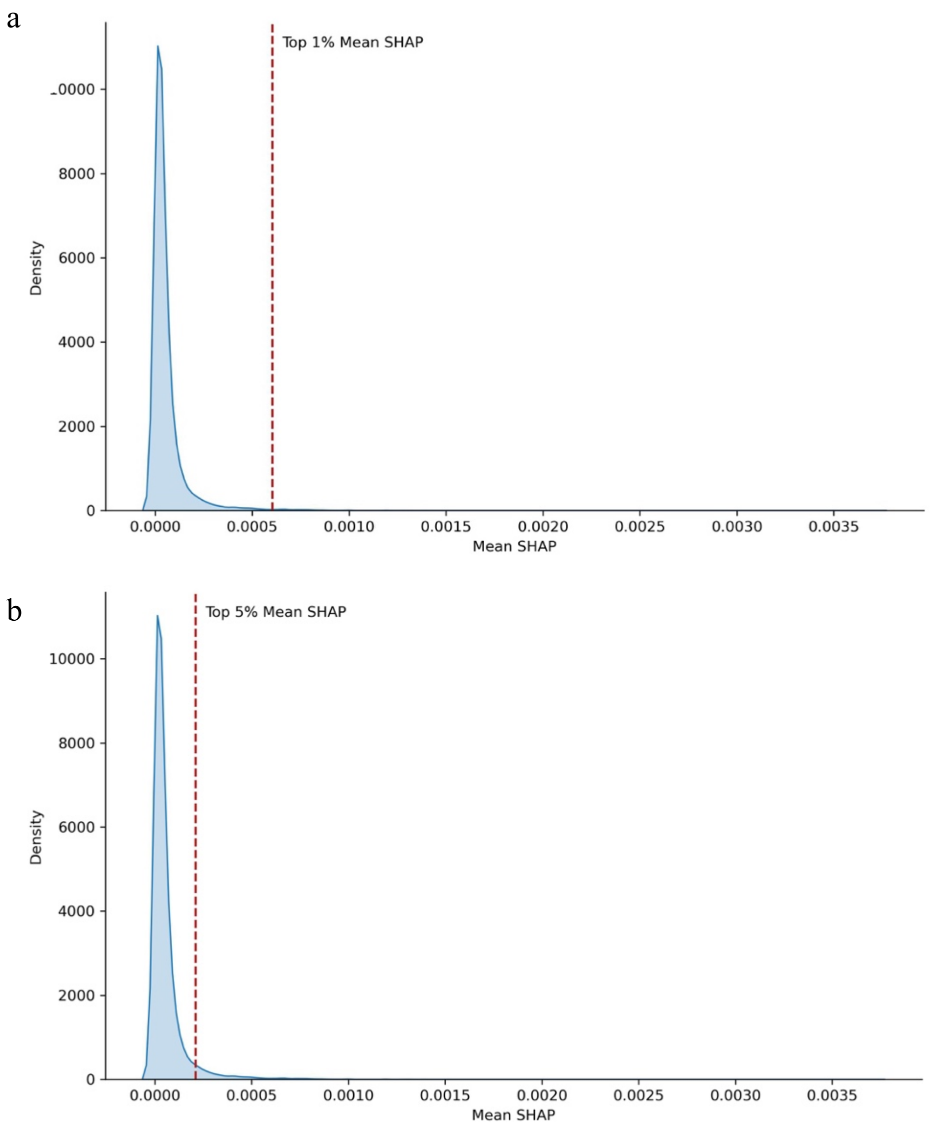

Figure S4: Distribution of mean absolute SHAP values for gradient boosting models trained with all SNPs, including the *APOE* region, showing (a) the top 1% threshold and a decrease in mean absolute SHAP values at around 0.0005 (~1.3% of the top SNPs), resulting in 108 SNPs selected, and (b) a looser 5% threshold which would have included a significantly larger number of variants. Visualisation shown for a single initial train-test split.

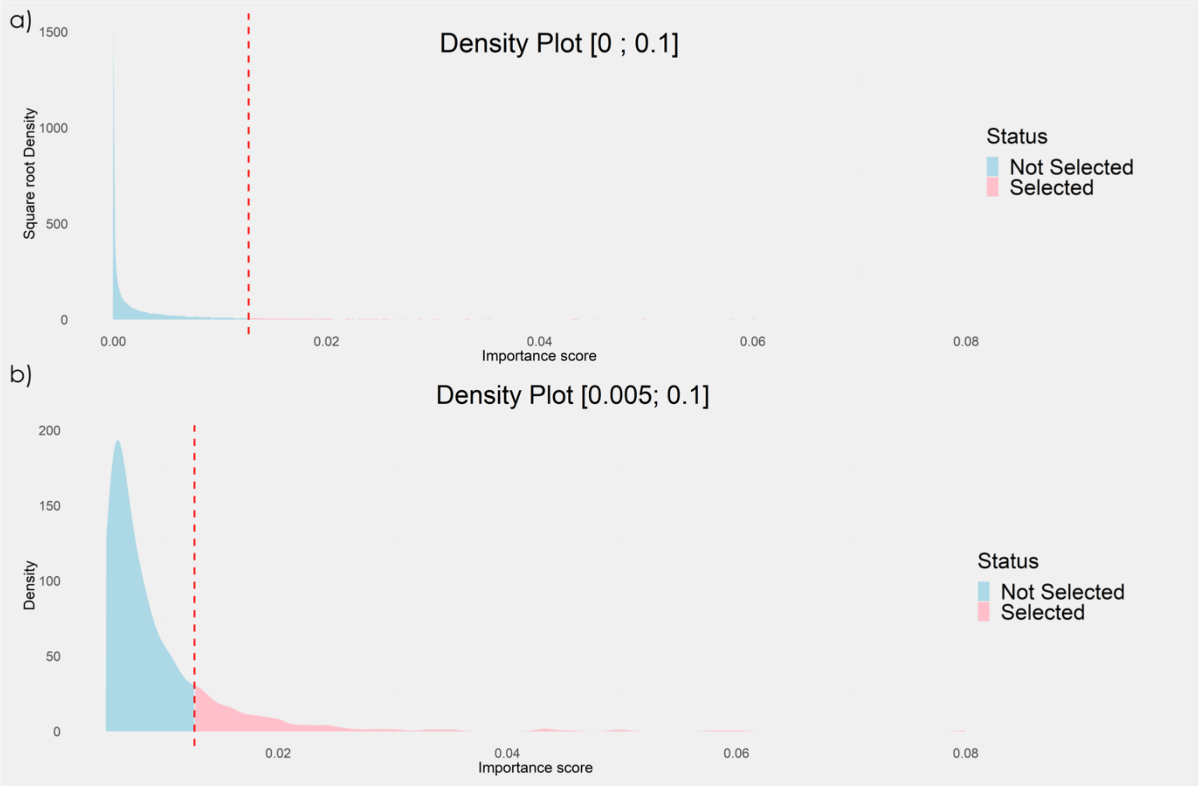

Figure S5: The distribution of importance scores for Neural Networks. The tail of the distribution was selected to give the extreme end of importance scores (137 SNPs selected). Scores are shown (a) as the full distribution, and (b) as a zoom plot to show the selected cut-off more clearly. Visualisation shown for a single initial train-test split.

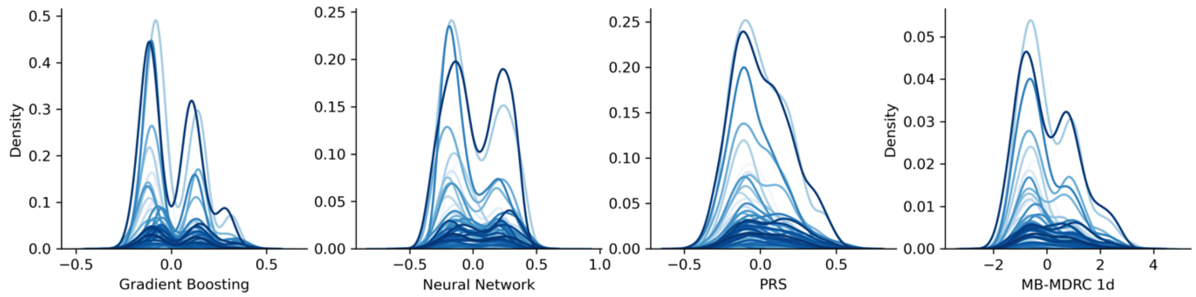

Figure S6: Distributions of adjusted model predictions from gradient boosting, neural networks, PRS and MBMDRC 1d trained on all SNPs, including the *APOE* region. Predictions from models show heavily overlapping multimodal distributions across constituent studies in the test split of the EADB-core, where peaks within a distribution are driven by *APOE* alleles. Distributions are coloured by the study within the EADB-core sample (legend not shown due to the large number of studies).

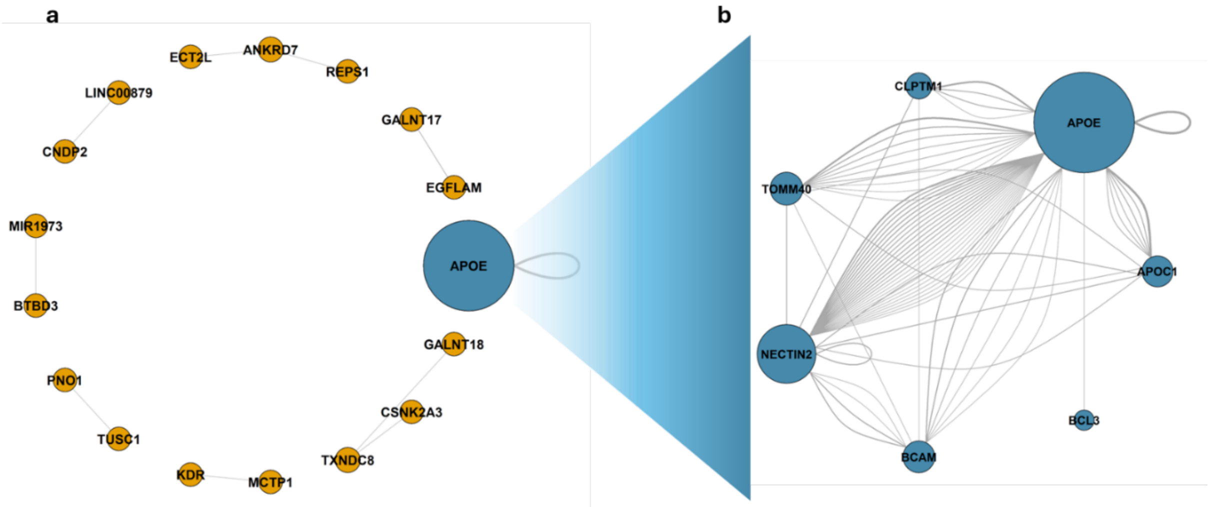

Figure S7: MB-MDRC 2d SNP-SNP interaction in a), with the node size proportional to the node degree. The same SNP can be mapped to multiple genes and thus the single SNP pairs can be shown as multiple gene pairs. In b), the SNPs in the APOE region are analyzed in detailed and mapped to the closest gene.

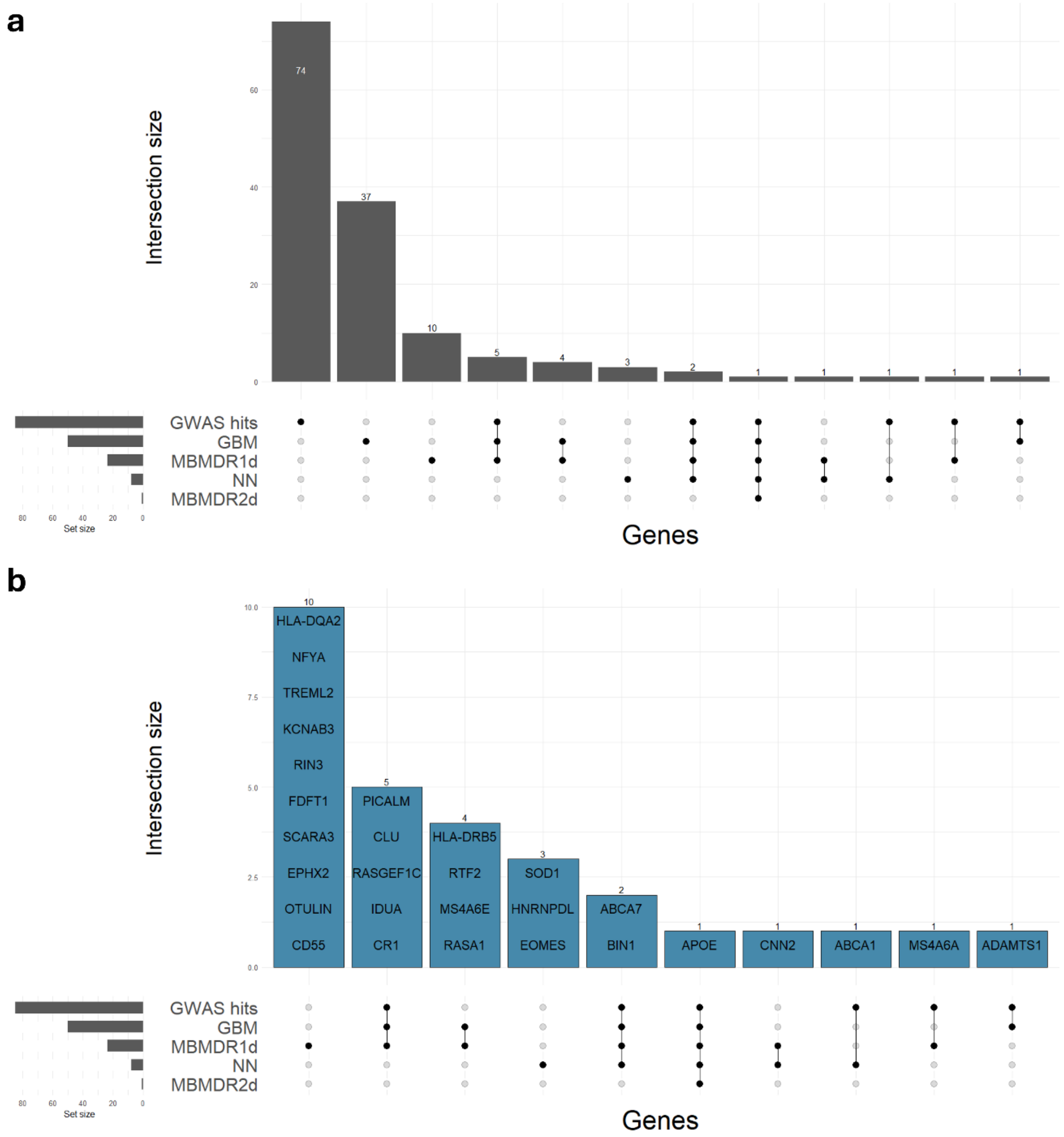

Figure S8: UpSet plot showing the overlap between ML and genome-wide significant findings from major AD GWAS (Supplementary Data 1). (a) Genes mapped by the SNPs highlighted by each ML approach separately. Both ML approaches and GWAS significant SNPs were identified in the same training split. (b) Genes that are shared among at least two train-test splits.

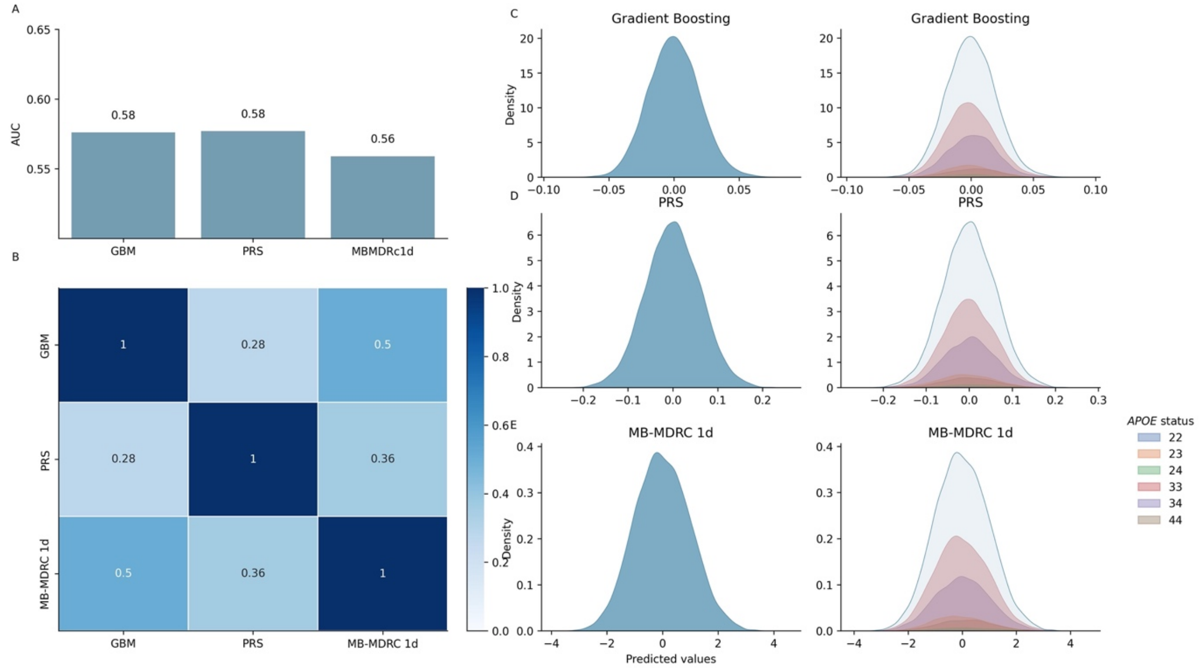

Figure S9: Test set predictions for models trained without the *APOE* region. Predictions are not significantly different between GBM and PRS (A); MB-MDRC 1d performed significantly worse than PRS (DeLong's test for two correlated ROC curves, p = 0.0018). Pairwise (Pearson's r) correlation show weak-to-moderate correlation between adjusted test set model predictions (B). Distributions of adjusted test-set predictions (C-E) do not differ by *APOE* status, as expected. Results for neural networks are not shown as prediction was not significantly better than chance. Prediction is only shown for the best-performing MB-MDR model (MB-MDRC 1d). All results are for a single initial train-test split; bars (A) represent a single value.

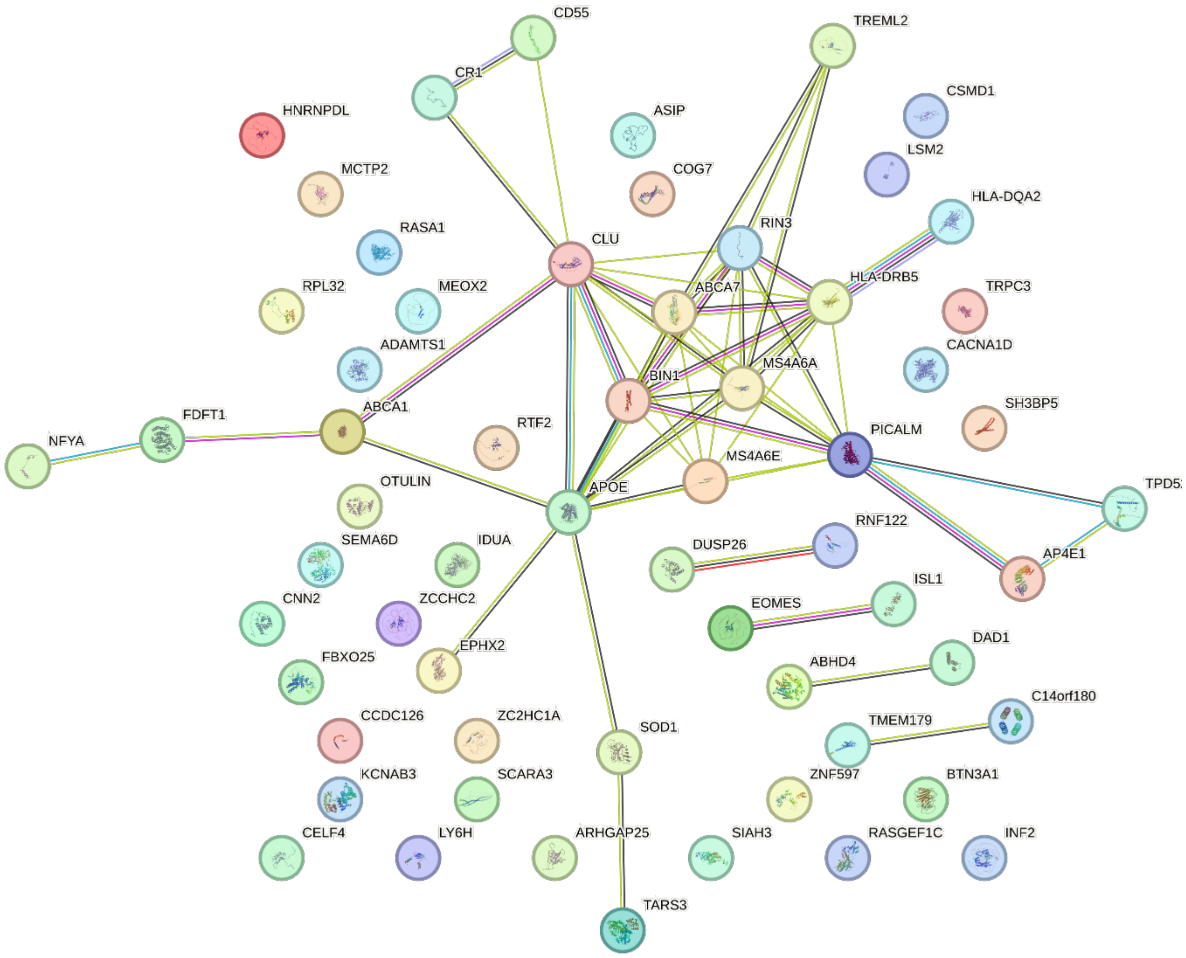

Figure S10: STRING networks for the 68 genes resulting from NNs, GBMs, MB-MDR 1d and MB-MDR 2d obtained with STRING. Only edges with moderate confidence (>=0.400, default setting) are shown. The line colour indicates the type of interaction evidence; known interactions are depicted in sky blue if from curated datasets, and violet if from experimentally determined data; predicted interactions are in green for gene neighbourhood, red for gene fusions and blue for gene co-occurrence. Other edges represent text mining (yellow), co-expression (black) and protein homology (grey).

Figure S11: A clustermap of scaled SHAP values from GBMs run on the four train-test splits (described in detail in Supplemental Material, Section 2.6). Each row is an individual and each column is a SNP, highlights groups of individuals and SNPs which are collectively impactful in predictions. Subplots A through D show the clustermap of SNPs from tables 1 and 2 which were present in the stability split.

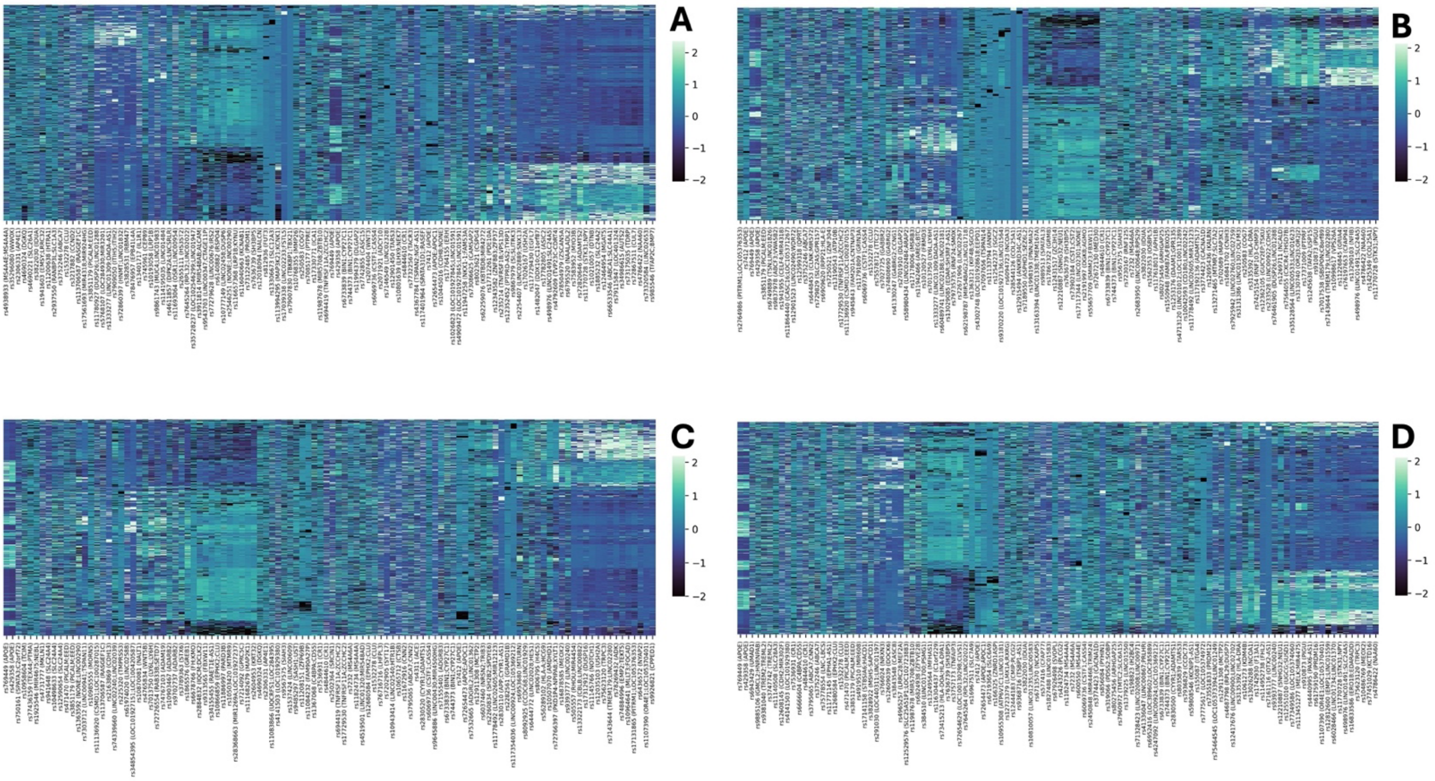

Supplement: Supplementary file 1 — Supplementary Information [file 41467_2025_61650_MOESM1_ESM.pdf]
