# Supplementary material for: Machine learning in Alzheimer’s disease genetics
Source: Nat Commun. 2025 Jul 22;16:6726. doi: 10.1038/s41467-025-61650-z (PMC12280214; doi:10.1038/s41467-025-61650-z)
Supplement: Supplementary file 2 — Description of Additional Supplementary Files [file 41467_2025_61650_MOESM2_ESM.pdf]

Description of Additional Supplementary Files

Supplementary Data 1: Previously reported genome-wide significant index variants. rs7412 (APOE e2 allele SNP) is not present in the Andrews/Wightman/Bellenguez lists, so not listed here, but is directly typed and present in the final QC'd dataset. "Reported gene" gives the gene/locus in previous publications. "Consensus gene" gives the annotations from the same positional (ensembl) and functional (OpenTargets Genetics) pipeline used to annotate ML hits to give a fair comparison. Note that "rs708382" (GRN, Wightman et al.) was excluded from the list of GWS SNPs to add from imputed data, but was already present in the genotyped data. It is therefore not "added" but is present in the final file

Supplementary Data 2: The architecture of the neural network model. Total params: 607,136; trainable params: 607,070; non-trainable params: 66.

Supplementary Data 3: P-value from DeLong's test for two correlated ROC curves. Calculated using pROC in R. AUCs for NN without APOE not shown as they are not significantly different from chance (0.5). Tests are calculated on AUCs from the initial train-test split only.

Supplementary Data 4: AUC comparison between ML models on different train-test splits.

Supplementary Data 5: Annotated hits from Machine Learning models. Genes at locus 18q12.2 are merged in the final Table 1.

Supplementary Data 6: Significant pairwise interaction test for all SNPs in Table 1 (main text). SNPs and covariates were both standardised (z-transformed) before regression models.

Supplementary Data 7: Pathway enrichment results (from PANTHER 19.0) from gene sets combining ML methods hits.
