# Supplementary material for: Machine learning in Alzheimer’s disease genetics
Source: Nat Commun. 2025 Jul 22;16:6726. doi: 10.1038/s41467-025-61650-z (PMC12280214; doi:10.1038/s41467-025-61650-z)
Supplement: Supplementary file 4 — Reporting Summary [file 41467_2025_61650_MOESM4_ESM.pdf]

nature portfolio

Corresponding author(s): Kristel van Steen
Cornelia van Duijn
Valentina Esscott-Price

Last updated by author(s): Jun 9, 2025

Reporting Summary

Nature Portfolio wishes to improve the reproducibility of the work that we publish. This form provides structure for consistency and transparency in reporting. For further information on Nature Portfolio policies, see our Editorial Policies and the Editorial Policy Checklist.

Statistics

For all statistical analyses, confirm that the following items are present in the figure legend, table legend, main text, or Methods section.

n/a	Confirmed	
☐	☒	The exact sample size (n) for each experimental group/condition, given as a discrete number and unit of measurement
☒	☐	A statement on whether measurements were taken from distinct samples or whether the same sample was measured repeatedly
☐	☒	The statistical test(s) used AND whether they are one- or two-sided<br>*Only common tests should be described solely by name; describe more complex techniques in the Methods section.*
☐	☒	A description of all covariates tested
☐	☒	A description of any assumptions or corrections, such as tests of normality and adjustment for multiple comparisons
☐	☒	A full description of the statistical parameters including central tendency (e.g. means) or other basic estimates (e.g. regression coefficient) AND variation (e.g. standard deviation) or associated estimates of uncertainty (e.g. confidence intervals)
☐	☒	For null hypothesis testing, the test statistic (e.g. F, t, r) with confidence intervals, effect sizes, degrees of freedom and P value noted<br>*Give P values as exact values whenever suitable.*
☒	☐	For Bayesian analysis, information on the choice of priors and Markov chain Monte Carlo settings
☒	☐	For hierarchical and complex designs, identification of the appropriate level for tests and full reporting of outcomes
☐	☒	Estimates of effect sizes (e.g. Cohen's d, Pearson's r), indicating how they were calculated

Our web collection on statistics for biologists contains articles on many of the points above.

Software and code

Policy information about availability of computer code

Data collection	No data collection and therefore software for it were used in this study.
Data analysis	Analysis used: shap 0.41, statsmodels 0.13.5, matplotlib 3.7.1, and seaborn 0.12.2, PLINK v2.00a3.3LM, the dask package, version 2023.1.1, LDAK version 5.2, bcftools version 1.14, XGBoost version 1.7.6, dbSNP build 156 39, ANNOVAR version 2020-06-07, Gencode v40, STRING v12.0, and ComplexUpset version 1.3.3, . Code for running biologically-informed neural networks via GenNet and Tensorflow is already published and available (https://github.com/ArnovanHilten/GenNet). Code used for running distributed gradient boosting using dask and XGBoost with covariate adjustment, preprocessing of genotypes, and applying distributed Boruta-SHAP feature selection has been made publicly available (https://github.com/seafloor/daxos). Pathway annotation and enrichment tests are also available on GitHub (https://github.com/seafloor/escott-price-lab-pipelines). We use MBMDR version 4.4.1 (http://bio3.giga.ulg.ac.be/index.php/software/mb-mdr).

For manuscripts utilizing custom algorithms or software that are central to the research but not yet described in published literature, software must be made available to editors and reviewers. We strongly encourage code deposition in a community repository (e.g. GitHub). See the Nature Portfolio guidelines for submitting code & software for further information.

Data

Policy information about availability of data

All manuscripts must include a data availability statement. This statement should provide the following information, where applicable:

- Accession codes, unique identifiers, or web links for publicly available datasets
- A description of any restrictions on data availability
- For clinical datasets or third party data, please ensure that the statement adheres to our policy

The primary EADB-core dataset may be accessed on application to the EADB consortium. Data for replicating analyses are publicly available for annotation (dbSNP build 156, ANNOVAR version 2020-06-07, Gencode v40, Open Targets Genetics), pathway analysis and protein-protein interaction (STRING v12.0), and bootstrap enrichment (microglia (44), astrocytes (45), synapses (46) - see manuscript reference numbers).

Research involving human participants, their data, or biological material

Policy information about studies with human participants or human data. See also policy information about sex, gender (identity/presentation), and sexual orientation and race, ethnicity and racism.

Reporting on sex and gender	The term sex is used exclusively following precedent set by a prior Nature Genetics publication on the same data (Bellenguez et al., 2022, https://doi.org/10.1038/s41588-022-01024-z) in which it was determined from genetic data, and we only refer to this term here. Sex was considered a potential confounder in the study design as proportions of individuals in sex categories may differ by cohort (and hence allele frequency and AD diagnosis). Overall, the sample is 59% female, with 62% female in cases and 57% in controls. Predictive performance was split by sex (AUC) on reviewer requests to demonstrate the effectiveness of deconfounding in machine learning models. Association results are not given by sex as it is adjusted for in analysis. Consent has not been given to share individual level data.
Reporting on race, ethnicity, or other socially relevant groupings	Socially constructed or relevant variables like race were not used in the manuscript.
Population characteristics	Median age at baseline (IQR): 73 (14), Diagnostic category is binary (1=present, 0=absent). 48% of the sample are cases. Genotype information is sensitive and cannot be shared.
Recruitment	Recruitment criteria differ by each cohort which was combined in the overall EADB-core sample. However, the case-control design used by constituent studies enforces a roughly equal weighting of cases and controls. More affected cases may be present where individuals are recruited through clinics rather than population screening, and the ratio of cases to controls will not reflect the underlying population of a region. Estimated predicted probabilities are therefore likely to be poorly calibrated and require adjustment for the base rate in a new population so as to not over-estimate the likelihood of an AD diagnosis if applied at the population level. Such a study design is, however, is well-suited to identifying associations as it is enriched for cases. This is how the data are utilised in the current study
Ethics oversight	Each constituent study was approved separately by relevant local boards to form the unified EADB-core dataset (see supplementary materials, Bellenguez et al., 2022, https://doi.org/10.1038/s41588-022-01024-z). The analyses and study here was approved by group leaders across the relevant data providers by the EADB Steering Committee.

Note that full information on the approval of the study protocol must also be provided in the manuscript.

Field-specific reporting

Please select the one below that is the best fit for your research. If you are not sure, read the appropriate sections before making your selection.

☒ Life sciences ☐ Behavioural & social sciences ☐ Ecological, evolutionary & environmental sciences

For a reference copy of the document with all sections, see nature.com/documents/nr-reporting-summary-flat.pdf

Life sciences study design

All studies must disclose on these points even when the disclosure is negative.

Sample size	Sample size for the particular analysis performed in this study was not pre-determined using a specific calculation. We have used all available data and there is no consensus for required sample size in the machine learning field.
Data exclusions	Data were excluded in a prior publication which describes the pre-processing of the EADB-core genotyping data used here (Bellenguez et al. "New insights into the genetic etiology of Alzheimer's disease and related dementias", Nat Genet 54, 412–436, 2022). These exclusions consisted of quality control procedures on both indi viduals and genotypes to ensure high-quality common variants from unrelated individuals were taken forward. In the current study, individuals were further excluded if they were all present the summary statistics used by Kunkle et al. ("Genetic meta-analysis of diagnosed Alzheimer's disease identifies new risk loci and implicates Aβ, tau, immunity and lipid processing", Nat Genet 51, 414–430, 2019), to ensure no overlap during clumping of genotypes. SNPs were also further excluded during clumping

	procedures (see manuscript), which served to reduce dimensionality and ensure variables were not collinear before modelling, and during minor allele frequency (MAF) thresholding, which ensured only common well-typed variants were used during analysis.
Replication	Findings were verified for stability internally and replicated externally. Internal stability checks were performed through repeated cross-validation, where only variants which were stable across repeats were retained. External replication was performed with these variants by verifying association in external summary statistics (without sample overlap with the current study). Variants present in the summary statistics at the 5% were considered replicated.
Randomization	N/A
Blinding	N/A

Reporting for specific materials, systems and methods

We require information from authors about some types of materials, experimental systems and methods used in many studies. Here, indicate whether each material, system or method listed is relevant to your study. If you are not sure if a list item applies to your research, read the appropriate section before selecting a response.

Materials & experimental systems

n/a	Involved in the study	
☒	☐	Antibodies
☒	☐	Eukaryotic cell lines
☒	☐	Palaeontology and archaeology
☒	☐	Animals and other organisms
☒	☐	Clinical data
☒	☐	Dual use research of concern
☒	☐	Plants

Methods

n/a	Involved in the study	
☒	☐	ChIP-seq
☒	☐	Flow cytometry
☒	☐	MRI-based neuroimaging

Plants

Seed stocks	N/a
Novel plant genotypes	N/a
Authentication	N/a
